# Memory Cells in Atopic Dermatitis: Paving the Way to Disease Modification

**DOI:** 10.3390/ijms27052371

**Published:** 2026-03-03

**Authors:** Raquel Dominguez-Lopez, Carlos J. Aranda, Enrique Gómez-de la Fuente, Bibiana Pérez-García, Javier Perez-Bootello, Carlota Abbad-Jaime de Aragon, Álvaro González-Cantero, Emilio Berna-Rico

**Affiliations:** 1 Department of Dermatology, Hospital Universitario Ramon y Cajal, Instituto Ramón y Cajal de Investigación Sanitarian (IRYCIS), 28034 Madrid, Spain; raquel.dom.31@gmail.com (R.D.-L.); egomezfu@gmail.com (E.G.-d.l.F.); bibianapg1@gmail.com (B.P.-G.); jpbootello@gmail.com (J.P.-B.); carlotababbad@gmail.com (C.A.-J.d.A.); alvarogc261893@hotmail.com (Á.G.-C.); 2 Department of Biochemistry and Molecular Biology II, School of Pharmacy, University of Granada, 18071 Granada, Spain; cjarandaclemente@gmail.com; 3 Institute of Nutrition and Food Technology “José Mataix” (INYTA), Centre of Biomedical Research, University of Granada, 18016 Granada, Spain; 4 Faculty of Medicine, Universidad Francisco de Vitoria, 28223 Madrid, Spain

**Keywords:** atopic dermatitis, immunological memory, tissue-resident memory T cells, CLA^+^ memory T cells, type 2 inflammation, memory B cells, epithelial alarmins, OX40/OX40L pathway, disease modification

## Abstract

Atopic dermatitis (AD) is a chronic relapsing inflammatory skin disease in which persistence of immunological memory underlies disease recurrence and progression toward atopic comorbidities. Evidence indicates that pathogenic tissue-resident memory T cells (TRM), including Th2- and Th22-skewed subsets, among others, persist in both lesional and clinically resolved skin and rapidly re-initiate inflammation through production of IL-4, IL-13, IL-22 and IL-31, promoting barrier dysfunction and pruritus. In parallel, circulating CLA^+^ memory T cells retain skin-homing capacity and contribute to flare reactivation, while IgG1^+^CD23 IL-4Rα^+^ type-2 memory B cells (MBC2) constitute a reservoir for high-affinity IgE production, linking cutaneous inflammation with allergic comorbidities. These adaptive memory compartments are sustained by epithelial alarmins, dendritic cell–derived chemokines such as CCL17, CCL22 and CCL18, and the OX40/OX40L costimulatory pathway, which promotes differentiation, survival and tissue retention of memory T cells. Clinical and transcriptomic studies show how, although IL-4/IL-13 blockade reduces circulating type-2 responses, Th2A cells, Tc2 cells and activated dendritic cells can persist in clinically resolved skin, providing a mechanistic basis for relapse after treatment withdrawal. Together, these findings support the relevance of targeting memory-imprinting pathways as a promising mechanism to achieve durable disease modification in AD.

## 1. Introduction

Atopic dermatitis (AD) is a chronic and relapsing inflammatory skin disease that affects individuals of all ages, with particularly high prevalence during childhood [1,2]. It is characterized by skin barrier dysfunction, dysbiosis, and predominantly Th2-driven immune-mediated inflammation [2]. The burden of AD on patients’ quality of life is significant: persistent itch, visible lesions, and frequent relapses contribute to sleep disturbances, psychosocial stress, school and work absenteeism, and overall deterioration of physical and emotional well-being [1]. Furthermore, AD, especially in moderate-to-severe forms, is frequently associated with comorbidities [3,4,5], particularly those of the atopic spectrum, such as food allergies, asthma, eosinophilic esophagitis, and allergic rhinitis [6,7]. The sequential emergence of these atopic comorbidities has traditionally been referred to as the "atopic march”, although this concept is currently a matter of debate [6,8]. All these factors make AD an important public health issue that demands adequate treatment. In this context, a deep understanding of its pathophysiology is essential to develop effective therapeutic strategies.

A key aspect in the chronicity and recurrence of AD is the role of adaptive immune memory cells [8,9,10], particularly memory T and B lymphocytes. These cells may persist in the skin and systemic circulation even after clinical remission, facilitating rapid and potent immune responses upon re-exposure to antigens [11]. Elucidating the mechanisms underlying memory cell differentiation and long-term survival is of great interest, since targeting these pathways may open the door to disease modification and help prevent progression to other atopic comorbidities [8].

In this review, we explore the role of cellular memory in AD, focusing primarily on adaptative immune memory, while also addressing the emerging concept of trained immunity within the innate immune system. We discuss how these memory programs contribute to pathogenesis, chronicity and relapses, as well as how current and emerging treatments may influence these cellular populations.

## 2. Adaptative Immune Memory Cells

Several subpopulations of memory T lymphocytes, including tissue-resident memory T cells (TRM) [10,11,12], particularly Th2A lymphocytes [13], and circulating cutaneous lymphocyte-associated antigen (CLA) + lymphocytes [9,14,15], have been identified as key players in the perpetuation of inflammation in AD. Furthermore, memory B cells, particularly those primed to switch to the production of high-affinity IgE, may play an important role in sustaining allergic responses (Table 1). Despite the success of new biological treatments targeting Th2 molecules, such as dupilumab, tralokinumab, lebrikizumab or nemolizumab, over 60% of patients do not achieve complete clearance after 16 weeks of therapy [16,17,18,19]. Furthermore, a significant proportion of patients who respond to these new therapies commonly experience disease relapses during treatment and after treatment withdrawal [20,21,22,23,24]. The mechanisms underlying non-response and relapses are not fully understood. Transcriptomic studies suggest that incomplete inhibition of distinct immune pathways and immunologic shunts may contribute to inadequate clinical control [25,26]. Furthermore, recent work has demonstrated that proinflammatory immune cell subsets persist even within clinically resolved skin after dupilumab treatment, indicating the presence of residual memory T cells that maintain a subclinical inflammatory state that may promote relapse upon treatment discontinuation [11].

## 3. Memory T Lymphocytes in Atopic Dermatitis

**Figure 1:**
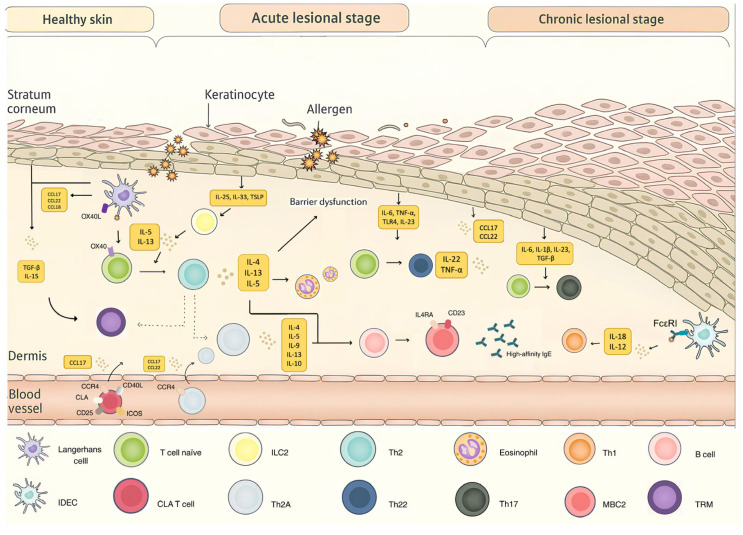
Modify from Weidinger et al. [34]. Damage to the cutaneous barrier allows antigens to penetrate and be captured by Langerhans cells, which subsequently present these antigens to naïve T cells. Under the influence of cytokines such as IL-5 and IL-13, naïve T cells differentiate into TH2 cells, which in turn secrete cytokines including IL-4, IL-13, and IL-5. These cytokines promote the recruitment of eosinophils and basophils, thereby exacerbating epidermal barrier dysfunction. Activated keratinocytes release cutaneous alarmins such as IL-25, IL-33, and TSLP, which act on skin-resident group 2 innate lymphoid cells (ILC2s). By secreting IL-5 and IL-13, these ILC2s not only enhance the differentiation of naïve T cells into TH2 cells but also amplify cutaneous inflammation. Activated keratinocytes also produce other cytokines such as IL-6, TNF-α, IL-23, and TGF-β, which induce T-cell differentiation toward TH22 and TH17 phenotypes. In addition, inflammatory dendritic epidermal cells (IDECs), through the expression of high-affinity IgE receptors (FcεRI) and the secretion of IL-18 and IL-12, promote TH1 differentiation. A proportion of activated effector T cells subsequently acquire a tissue-resident memory T-cell (TRM) phenotype. Their long-term maintenance depends on local secretion of TGF-β and IL-15 by keratinocytes and macrophages. Among these TRM cells is a specialized subset, the TH2A cells, which produce IL-4, IL-5, IL-9, IL-13, and IL-10. These cytokines stimulate B cells, some of which differentiate into MBC2 memory B cells, characterized by expression of ILR4 and CD23, and responsible for generating high-affinity IgE. In the peripheral circulation, CLA-positive T cells expressing cutaneous lymphocyte antigen (CLA), CD25, CD40, and ICOS migrate toward the skin via cutaneous homing receptors. TH2A cells may also be present in peripheral blood and are recruited to the skin through receptors such as CCR4. CCL17 released by keratinocytes and APCs binds to CCR4, promoting the migration of Th2 lymphocytes from the peripheral circulation into the skin. Created with Canva.

T lymphocytes play a central role in the pathophysiology of AD, acting as key drivers in both the acute and chronic phases of the disease [2,27] (Figure 1). The adaptive response in AD begins with the activation of allergen-specific CD4+ T cells by antigen-presenting cells (APCs), which triggers a cascade of Th2 cytokine production (IL-4, IL-13, IL-5 and IL-31, among others) [2]. These cytokines promote the recruitment of other cell types, such as eosinophils, basophils and mastocytes, thereby amplifying inflammation, impair skin barrier function, and promote both pruritus and percutaneous sensitizations [2,28,29]. While Th2 cells have traditionally been considered the principal drivers of AD, other subsets, such as Th22, Th17 and Th1 cells, also participate, particularly in later or chronic stages of the disease [30,31,32]. After the effector phase, a proportion of T cells persist as memory cells, and their role in perpetuating inflammation and clinical relapses has become increasingly recognized [11,33].

The CLA is an adhesion molecule predominantly expressed on memory T lymphocytes with skin-homing properties, although these cells can also be detected in the blood and lymphoid tissues. CLA^+^ T cells, including both CD4^+^ and CD8^+^ subsets, are key mediators of cutaneous immune responses, and their frequency in peripheral blood correlates with disease activity and clinical severity in AD [15]. Consequently, they have been proposed as minimally invasive peripheral biomarkers. While naïve T cells do not express CLA, its expression is induced during the transition from a naïve to a memory phenotype [35]. CCL17 drives the recruitment of CLA^+^ T cells to the skin through binding to CCR4 expressed on these cells [1,2,14]. Although initially thought to be restricted to effector CD45RO^+^CCR7^−^ cells, it is now recognized that CLA expression is present across all memory T-cell compartments, underscoring its fundamental role in skin-homing immune memory [1,14].

CLA+ T cells in patients with AD exhibit an activated phenotype, characterized by high expression of HLA-DR, CD25, ICOS, and CD40L, and secrete multiple proinflammatory cytokines, predominantly Th2-type (IL-4, IL-5, IL-13 and IL-31), but also IL-17, IL-22, IFN-γ, and TNF-α, depending on their specific immune polarization. Thus, these cells contribute to neurogenic inflammation (via IL-31), induce IgE production by B cells, and promote eosinophil survival [9,14,15]. The number of CLA+ Th22 cells increases with age in AD patients and correlates with disease severity, pruritus, and IL-17 levels. Conversely, CLA+ Th1 cells are reduced, suggesting a persistent Th2/Th1 imbalance [1,9,15].

Treatment with dupilumab has been shown to reduce circulating CLA+ T cells producing Th2 cytokines, a finding that may be related to an indirect decrease in TRM and could theoretically favor longer remission periods [36]. However, although remission-free intervals appear longer with dupilumab than with JAK inhibitors, current evidence remains limited, and additional mechanistic and clinical data are needed to confirm these hypothesis and observations [23,24]. In this regard, drugs targeting OX40, a receptor highly expressed on CLA+CD4+CD45RO+ cells, are particularly promising for their hypothetical potential to disrupt the generation and persistence of this population [37].

### 3.1. Tissue-Resident Memory T Cells (TRM)

Tissue-resident memory T cells (TRM) represent a non-recirculating subset of memory T cells that remain localized within peripheral tissues, including the skin [38]. Their presence has been demonstrated not only in active lesions but also in clinically resolved skin, suggesting their role in maintenance and recurrence following treatment discontinuation [10,11,12]. Therefore, TRM are considered a promising therapeutic target for strategies aimed at achieving more durable remissions.

**Figure 2:**
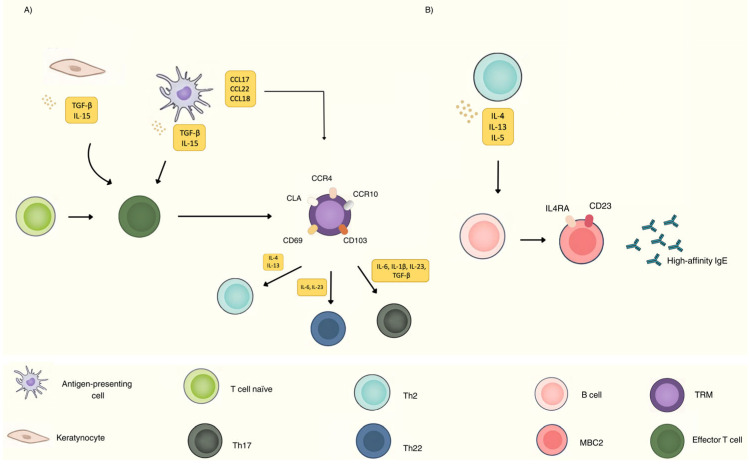
(**A**) Keratinocytes and antigen-presenting cells secrete TGF-β and IL-15 that act on activated effector T cells, promoting their acquisition and long-term maintenance of a tissue-resident memory T-cell (TRM) phenotype. TRM comprise functionally polarized subsets, including Th2 cells under the influence of IL-4 and IL-13, Th22 cells driven by IL-6 and IL-23, and Th17 cells induced by IL-6, IL-1β, IL-23, and TGF-β. Antigen-presenting cells also produce chemokines such as CCL17, CCL22, and CCL18, which contribute to the retention and persistence of T cells within the cutaneous microenvironment. (**B**) A Th2 cell secretes IL-4, IL-13 and IL-5, which act on B cells to induce their differentiation into MBC2 expressing IL4RA α and CD23 on their surface. These MBC2 serve as precursors of high-affinity IgE-producing plasma cells, thereby sustaining pathogenic humoral immunity in atopic dermatitis. Created with Canva.

Cutaneous TRM express a specialized phenotypic profile that includes CLA, chemokine receptors (CCR4, CCR10), and tissue retention molecules (CD69, CD103), as well as signaling pathways associated with survival and metabolic adaptation to the epidermal microenvironment (IL-15, mTOR, oxidative fatty acid metabolism). Cytokines such as IL-15, which is produced by keratinocytes, APCs, and fibroblasts [39], and TGF-β, which is expressed by a wide spectrum of cell types including keratinocytes, fibroblasts, macrophages and endothelial cells [40,41,42] play a critical role in the survival and retention of TRM in the skin [10,12,43] (Figure 2A).

Available transcriptomic evidence is consistent with a model in which activated effector T cells acquire transcriptional programs compatible with tissue residency and long-term persistence within the inflamed skin microenvironment. Single-cell RNA sequencing studies of lesional skin have identified T-cell subsets displaying combined signatures of effector activation and memory-associated gene expression, supporting the plausibility of an effector-to-TRM transition within the tissue context [33,44]. Moreover, type 2–polarizing signals such as IL-4 during T-cell priming have been shown to influence long-term memory programming and functional stabilization of differentiated T cells [45].

These stable transcriptional programs are likely reinforced by epigenetic mechanisms that govern TRM differentiation and long-term persistence. In particular, dynamic DNA methylation has been shown to regulate memory T-cell fate, with DNMT3A-mediated de novo methylation consolidating effector commitment, while memory-precursor cells retain the ability to partially erase these marks and re-establish durable memory programs [46]. In AD, the persistence of IL-4–, IL-13–, and IL-22–producing TRM populations supports the existence of stable epigenetic imprinting, including stable DNA methylation changes and locus-specific demethylation at effector genes that enable rapid recall responses in memory T cells, thereby sustaining cutaneous inflammatory memory and contributing to disease relapse [11,15,47,48,49]. Accordingly, targeting epigenetic regulators such as DNMT3A may represent a strategy aimed at long-term immune reprogramming rather than purely symptomatic control.

In AD, TRM encompass multiple subtypes, including CD4^+^ T helper cells (Th2, Th22, Th17) and CD8^+^ cytotoxic T cells (Tc2, Tc22, Tc17). Since type 2 immunity predominates in both acute and chronic lesions of AD, Th2/Tc2 memory cells may be particularly relevant [11,44]. Chemokines such as CCL17 and CCL22, produced by dendritic cells, macrophagues and keratinocytes, among others [50,51], may contribute to the differentiation and cutaneous recruitment of these Th2/Tc2 memory cells, as their corresponding receptors are expressed by them [11]. In addition, CCL18, also expressed by dendritic cells and macrophagues, has emerged as a relevant mediator of skin homing, acting as a chemoattractant for memory T cells and thereby further supporting the recruitment and persistence of memory populations within inflamed atopic skin [52,53,54]. As part of the Th2 response, these cells produce IL-4 and IL-13, among other cytokines. Several studies have suggested that IL-4 promotes the long-term maintenance of CD8^+^ T cells. Nevertheless, whether IL-4 and IL-13 regulates the generation and/or maintenance of TRM cells in the skin of patients with AD is still not completely understood [45,55,56,57]. Therefore, the notion that monoclonal antibodies targeting the IL-4/IL-13 pathway (dupilumab, tralokinumab, lebrikizumab and eblasakimab) may reduce the number or function of type 2-skewed TRM cells remains purely speculative, as studies specifically addressing this question are very limited. Adding further uncertainty, a recent study have showed how these cells may persist in the skin of AD patients even after one year of successful dupilumab therapy [11]. Exploring other Th2 cytokines may thus be relevant in this context. In this regard, recent evidence suggests that IL-9 may further promote the pathogenic function of Th2 TRM by upregulating IL-18 receptor expression through the IL-9R–JAK1/JAK3-STAT1 pathway, thereby sensitizing these cells to IL-18 signals and amplifying their secretion of proinflammatory cytokines such as IL-13 and IL-22 [58]. Exploring whether therapeutic strategies targeting IL-9 or IL-18 could affect the survival and pathogenicity of these memory cells represents an area of high clinical interest.

Within type 2-skewed TRMs, Th2A lymphocytes are a specialized subset of CD4+ memory T cells characterized by the CRTH2+CD161+ST2+CD27− phenotype, which includes allergen-specific Th2 cells in atopic individuals [11]. Signals that promote their differentiation, activation, and maintenance may include the classic epidermal alarmins (IL-25, IL-33, and TSLP) secreted by keratinocytes, as the presence of their receptors on the surface of these cells (IL17RB, ST2, and CRLF2, respectively) has been demonstrated [11,59]. Th2A lymphocytes are capable of producing a broad repertoire of cytokines essential to allergic responses (IL-4, IL-5, IL-9, IL-13, IL-10), and their abundance correlates with clinical severity in diseases such as AD, asthma, and food allergy [13]. In AD, Th2A cells can be found in both in lesional and non-lesional skin, including areas with resolved eczema [11,59]. They are the only cells that simultaneously produce IL-5, IL-13, and IL-31, the latter two being directly involved in pruritus development, and are absent in healthy skin, reinforcing their pathogenic role [13].

Th22 polarization represents another key immune pathway in the immunopathogenesis of AD. The progressive barrier dysfunction and dysbiosis driven by Th2 cytokines, together with mechanical injury from itch and scratching, further amplify keratinocyte activation. Activated keratinocytes not only secrete classic epidermal alarmins such as TSLP, IL-25 and IL-33, but also IL-6, TNF- α, Toll-like receptor 4 (TLR4) ligands and IL-23 [31,60,61], which, together with IL-23-stimulated dermal dendritic cells, promote Th22 differentiation [62,63]. These Th22 cells produce IL-22 and TNF-α but not IL-17, contributing to epidermal hyperplasia, barrier dysfunction, and pruritus [32]. IL-6, IL-1β, IL-23, and TGF-β, are other cytokines produced by activated keratinocytes and eosinophils within the inflammatory AD milieu, driving Th0 to Th17 differentiation [30,31]. Although less dominant than Th2 pathways, Th17 responses are particularly relevant in certain AD phenotypes, such as in Asiatic people [64], contributing to barrier impairment, antimicrobial peptide induction, and sustained inflammation [30,31]. Despite these observations, much of the evidence supporting the presence and relevance of Th22 and Th17 responses in AD derives from transcriptomic studies describing cytokine expression rather than from direct cellular characterization [25,47,64]. Direct phenotypic and functional profiling of these cellular populations using single-cell and flow-cytometry approaches remains very limited [11,33,44,65].

In summary, TRM populations in AD are still not well characterized, and emerging data reveal considerable heterogeneity and broad cytokine-producing capacity [44]. Their possible expansion in response to repeated epithelial triggers supports their contribution to chronicity and relapses, underscoring the need for more detailed mechanistic studies to clarify their role and therapeutic relevance.

### 3.2. Regulatory T Cells and Their Interplay with Immune Memory in Atopic Dermatitis

Regulatory T cells (Tregs) represent an immune cell population of increasing interest in AD, although their specific role in the regulation of cutaneous immune memory remains incompletely defined [66,67]. Beyond their classical function in maintaining immune tolerance, Tregs are involved in the control of memory T cell-mediated responses [68]. In AD, Tregs have been reported to exhibit functional alterations and, in some contexts, to acquire a dysfunctional or proinflammatory phenotype with impaired suppressive capacity, thereby contributing to the persistence of chronic inflammation [66,69]. This imbalance is further promoted by the Th2-skewed cytokine milieu characteristic of the disease, as interleukin-4 inhibits the differentiation of naïve T cells into Tregs, disrupting the balance between regulatory and effector mechanisms [70,71].

Under physiological conditions, functional Tregs contribute to tissue immune homeostasis by restraining the activation and persistence of TRM [67,68]; however, in AD this regulatory interaction appears to be compromised [69]. Notably, recent studies using selective interleukin-2 receptor pathway agonists, such as rezpegaldesleukin, have demonstrated Treg expansion associated with a reduction in interleukin-15 levels, a cytokine essential for the maintenance and survival of TRM, as previously discussed [12,72,73]. Although this field remains at an early stage of investigation, these findings support the hypothesis that disruption of the Treg–IL-15–TRM axis may contribute to disease chronicity and relapse in AD, and suggest that restoration of regulatory function could represent a future disease-modifying strategy [72].

## 4. Memory B Lymphocytes in Atopic Dermatitis

In AD, B lymphocytes participate in the adaptive immune response by producing IgE. This production is driven by Th2 cytokines, primarily IL-4 and IL-13, which induce class switching and expansion in naïve and memory B cells [74]. Upon activation, B cells may differentiate into short-life plasmablasts, or undergo affinity maturation within germinal centers and subsequently exit as memory B cells or fully differentiated IgE-producing plasma cells [2,75].

In contrast to other isotypes, it has been accepted that IgE+ memory B lymphocytes are extremely low in number, to the point that their existence has long been questioned [76]. Studies in mice and human have found transient memory IgE cells that disappear shortly, usually by apoptosis [74,75]. However, recent studies have challenged this paradigm, showing that IgE-specific immunological memory is mainly maintained in IgG1+ memory B cells, particularly those with the CD23hi IL-4Rα+ phenotype, referred to as type 2 memory B cells (MBC2) (Table 1). These cells exhibit active transcription of the germline IGHE locus, positioning them as precursors of high-affinity IgE-producing plasma cells [74,75,77,78] (Figure 2B).

MBC2s are increased in atopic individuals, and their frequency correlates positively with total serum IgE levels. These cells are believed to act as a long-term reservoir of atopic humoral memory, with a predisposition to class switching to IgE upon antigen re-stimulation [74]. This molecular profile includes overexpression of IL4R, IgG1^+^ and FCER2 (CD23), rendering them highly reactive to Th2 stimuli, such as IL-4 or IL-13 [77]. In a recent clinical study in a pediatric population, treatment with dupilumab was associated with a significant decrease in circulating MBC2 cells and a reduction in total IgE. This finding suggests that therapies targeting IL-4/IL-13 signaling could directly interfere with the persistence and reactivation of memory B cells associated with pathogenic IgE production [78].

However, the specific contribution of MBC2 cells to total IgE production in AD remains incompletely understood. It is estimated that, in this disease, a portion of circulating IgE may arise from direct class switch recombination (CSR) from IgM/IgD to IgE, resulting in low-affinity IgE by short life plasmablasts [75]. This contrasts with high-affinity IgE generated through sequential CSR involving IgG1^+^ memory cells. Therefore, changes in MBC2 cell numbers may only partially explain the effects of anti-IL-4Rα therapies on IgE levels, given the contribution of alternative IgE-producing pathways in atopic dermatitis [78].

Furthermore, the direct pathogenic role of IgE in AD remains controversial. Clinical evidence, however, indicates that percutaneous exposure to allergens to which the patient is IgE-sensitized can trigger disease exacerbation with a delayed onset [79], a pattern suggestive of a type IV reaction. The binding of the allergen to IgE, which interacts with high-affinity IgE receptors (FcεRI) on Langerhans cells and other APCs, lead to antigen internalization and the subsequent antigen presentation to T lymphocytes, which initiates and amplifies the inflammatory cascade [60]. This type IV reaction through FcεRI could also be involved in the sequential appearance of Th1 lymphocytes in AD lesions, as Novak et al. demonstrated that FcεRI engagement on inflammatory dendritic epidermal cells (IDECs) induces the production of proinflammatory cytokines such as IL-18 and IL-12, which in turn promote the differentiation of IFN-γ-producing Th1 cells [80].

Finally, there is evidence suggesting that the development of food or environmental sensitizations may occur percutaneously in the context of AD itself [81], as skin barrier dysfunction facilitates allergen penetration in an immunological environment characterized by strong Th2 cytokine overexpression (IL-4, IL-13, IL-9) [82], thereby favoring B-cell activation and production of antigen-specific IgE, and the development of memory B-cells populations, including MBC2 [61]. This revives the classical concept of the atopic march. The possibility of inhibiting the reactivation or differentiation of these cells into IgE^+^ plasma cells through targeted immunomodulation could thus potentially prevent the progression of AD into other atopic comorbidities and thereby alter its natural course, as the presence of such comorbidities is associated with more severe and persistent disease [8,81].

## 5. Influence of Innate Immunity on Adaptative Immunity

The innate immune system critically shapes the adaptive responses that sustain long-term immunological memory in AD. Epidermal barrier dysfunction—whether genetically determined or environmentally induced—drives the persistent release of epithelial alarmins, which maintain low-grade activation of key innate sentinels such as keratinocytes, Langerhans cells (LCs), inflammatory dendritic epidermal cells (IDECs), and group-2 innate lymphoid cells (ILC2s) [11,59,82] (Table 1). This alarmin-rich milieu promotes early Th2 polarization, supports survival of Th2-skewed TRM populations, and facilitates rapid reactivation upon barrier disruption [83,84].

LCs occupy a central position at this innate–adaptive interface. As epidermal antigen-presenting cells with high expression of Langerin/CD207, they survey the skin surface, capture allergens through tight junctions, and migrate to draining lymph nodes to prime T cells [85]. LCs can polarize naïve T cells toward Th2, Th22, or Th17 fates depending on context, and their activation by TSLP or IgE–FcεRI ligation enhances the recruitment and expansion of Th2 cells [30,60,80,83,85,86,87]. In AD, LCs display increased activation and maturation and accumulate in lesional skin, supporting their contribution to the persistent Th2 imprinting that underlies memory responses [85]. IDECs, a related FcεRI^+^ dendritic subset, amplify this process by efficiently capturing IgE–allergen complexes and promoting both Th2 responses and the Th1 skew characteristic of chronic AD [60,80,83,87].

Innate lymphoid cells add an additional layer to memory formation. ILC2s respond rapidly to alarmins (TSLP, IL-33, IL-25, IL-18) by producing IL-5, IL-13, and amphiregulin, thereby reinforcing early Th2 polarization and shaping an environment that facilitates the establishment and maintenance of Th2-type TRM [65,88,89,90,91]. Their expression of OX40L further enhances local Th2 adaptive immunity [12]. Although less directly involved, ILC3s produce IL-17 in response to IL-1 and IL-23 from activated keratinocytes, indirectly strengthening type 2 circuits and illustrating the plasticity of innate responses in AD [92].

Beyond immediate activation, innate cells may also contribute to longer-term immune memory. The persistence of IL-15-producing dendritic cell subsets after clinical remission suggests a form of “innate tissue memory” capable of supporting survival of TRM populations [11]. In parallel, the emerging concept of trained immunity—epigenetically driven reprogramming of innate cells—raises the possibility that repeated epithelial injury or dysbiosis could imprint monocytes and dendritic cells with a durable Th2-promoting phenotype, although this remains largely unexplored in AD [88,93].

Collectively, these pathways illustrate how innate immune mechanisms not only initiate AD inflammation but also imprint, sustain, and reactivate adaptive immune memory, thereby contributing to chronicity and relapse [90,91].

### 5.1. The OX40/OX40L Pathway in Atopic Dermatitis

The OX40 (CD134)/OX40L (CD252) signaling axis constitutes a key immunological pathway in the expansion, survival, and differentiation of effector and memory T cells [27,94]. OX40 is a transiently expressed co-stimulatory receptor on activated CD4^+^ T lymphocytes, while its ligand, OX40L, is expressed on APCs, ILC2s, keratinocytes, mast cells, and other immune cells, thus representing a key link between innate and adaptative immunity. This pathway is particularly relevant in AD due to its pivotal role in maintaining type 2 inflammation and generating pathogenic memory T cells [27,95].

#### 5.1.1. Activation and Functions of the OX40/OX40L Pathway

Following initial activation of naïve T cells by APCs, the expression of OX40 and OX40L is induced between days 1 and 5, establishing a secondary co-stimulatory signal. This interaction enhances T cell proliferation, prolongs survival, and induces differentiation toward Th2, Th17, and Th22 subsets through sustained cytokine production, including IL-4, IL-5, IL-13, IL-17, IL-22, and IL-31 [94].

TSLP expressed by keratinocytes is particularly relevant in this context, as it induces OX40L expression on dermal dendritic cells even in the absence of IL-12, thereby promoting the polarization of naïve T lymphocytes toward a Th2 phenotype [83]. OX40 functions as a general co-stimulatory molecule—not exclusive to Th2 cells—but in this context, the absence of IL-12 prevents the development of other T cell subsets, consolidating an early Th2 phenotype [27]. Subsequently, IDECs infiltrate the skin and, upon antigen uptake via their FcεR1, begin producing IL-12, as stated before, thereby promoting the differentiation of additional T cell lineages [83,84]. Furthermore, polymorphisms in the TNFSF4 gene, which encodes OX40L, have been associated with increased susceptibility to AD, which also highlights the pathophysiological relevance of this pathway [95].

#### 5.1.2. Role in Immunological Memory and Chronicity

Persistent activation of the OX40/OX40L pathway promotes not only effector responses but also the generation of memory T cell subsets. Experimental models and human studies have shown that this pathway directly contributes to chronic skin lesions, inflammation perpetuation via memory cells, and the transition from acute to chronic disease stages [27,95].

Cells expressing OX40L are increased in both lesional and non-lesional skin of AD patients [94,95], suggesting an underlying inflammatory state and persistent immunological “imprinting” even in the absence of active clinical disease [27,95]. Moreover, OX40 signaling has been shown to facilitate the conversion of activated T cells into long-lived TRM, supporting their retention and persistence within the skin [95]. This may explain the occurrence of spontaneous flares or flare-ups triggered by minimal stimuli in previously affected areas.

#### 5.1.3. Therapeutic Implications

The OX40/OX40L axis represents an attractive therapeutic target due to its specificity for activated T cells, avoiding broad immunosuppression. Since OX40 is not expressed on naïve or resting T cells, its inhibition may selectively block antigen-specific effector and memory cells responsible for AD persistence [27,95].

Several monoclonal antibodies targeting this pathway—such as amlitelimab, telazorlimab, and rocatinlimab—are currently in development, with preliminary results showing promising clinical efficacy and safety profiles [96,97,98]. Therefore, these agents may not only control disease flares but also induce sustained remission by directly targeting the memory cell reservoir [99]. Moreover, blocking the OX40/OX40L pathway may modulate multiple inflammatory axes simultaneously (Th2, Th17, Th22), offering a more comprehensive therapeutic approach in AD patients with mixed inflammatory profiles [94].

## 6. Disease Modification in Atopic Dermatitis

The concept of disease modification refers to the ability of a therapeutic intervention to durably alter the natural course of a disease beyond mere symptomatic control. According to the FDA definition, a disease-modifying treatment exerts a lasting effect on the underlying pathophysiology—even after discontinuation of the drug—by reducing disease incidence, severity, or recurrence [100,101]. This approach implies a clinical trajectory change with sustained long-term impact, as well as modification of associated comorbidities, helping reduce their occurrence or severity [8]. In AD, this concept is gaining increasing relevance in light of recent advances elucidating the chronic immunological mechanisms underlying the disease, particularly the contribution of memory-competent immune cell populations.

A central question is whether early and proactive intervention can change the course of AD, reducing flare-ups, severity, or the development of other atopic comorbidities. This could provide indirect evidence for strategies that target these memory cells. Several studies suggest that early intervention may mitigate key immunological processes such as IgE sensitization, this reducing the risk of developing atopic comorbidities [8]. For instance, in a pediatric study, proactive rather than reactive topical corticosteroid treatment improved clinical outcomes and reduced allergic sensitization, a major predictor of poor AD prognosis [102]. Moreover, in a large population-based cohort study, pediatric patients treated with dupilumab had lower risk of developing asthma and allergic rhinitis compared to those receiving conventional immunomodulatory therapies [103]. This evidence supports the existence of “therapeutic windows” in which cellular memory pathways may be particularly amenable to intervention, with potential implications for secondary prevention strategies.

Another dimension of disease modification concerns whether available therapies can maintain disease control after cessation. Rocatinlimab has shown sustained benefits for up to 20 weeks post-treatment [96], with 73–96% of EASI75 responders at Week 36 remaining relapse-free at Week 56 (equating to 20 weeks post-last dose at Week 36). Similar prolonged responses have been reported for lebrikizumab [21,104,105]. These observations raise the possibility that modulation of memory-competent immune populations may underlie these extended remissions. However, mechanistic evidence remains limited and partially contradictory—as highlighted by findings with dupilumab [11]—and no therapy for AD has yet been officially classified as disease-modifying by regulatory agencies.

Within this context, the concept of super responders (SRs) offers an additional lens through which to explore disease modification. SRs are patients who achieve rapid, profound, and sustained improvements in both clinical and patient-reported outcomes—typically within weeks 16–24 and maintained through Week 52 [106]. Evidence from psoriasis supports the relevance of this construct: studies such as GUIDE demonstrated that early and profound responses (PASI100) are associated with durable disease control [107], reinforcing the idea that identifying SRs can reveal windows in which immunological pathways are more modifiable.

The JADE DARE trial showed that abrocitinib was associated with a faster and more frequent attainment of super-response than dupilumab, particularly among female patients and those without prior cyclosporine exposure [106,108]. Additionally, in a prospective observational Spanish study, 55.6% of patients with moderate-to-severe AD treated with tralokinumab were considered SRs [109]. Identifying SRs is relevant not only for personalizing therapy but also for understanding which patients may achieve durable immunological recalibration. SRs may represent individuals in whom memory-imprinted pathways are more susceptible to modification, offering indirect insights into when and in whom disease modification could realistically be achieved. Yet, a unified definition of SRs and robust baseline predictors are still lacking, underscoring the need for harmonized criteria and mechanistic studies [110].

Advances in cellular immunoprofiling and high-throughput molecular tools are now clarifying the immune circuits that sustain disease memory. These insights pave the way toward a future in which the therapeutic goal is not merely flare control but induction of stable immune tolerance, thereby breaking the cycle of inflammation and relapse that defines AD.

## 7. Conclusions

Atopic dermatitis (AD) is a chronic immune-mediated disease in which persistent immunological memory—driven by pathogenic T and B cell subsets such as TRM (particularly Th2A), circulating CLA^+^ T cells, and IgG1^+^ memory B cells—underlies relapse and contributes to atopic comorbidities. Even after clinical improvement, these populations can persist and sustain subclinical inflammation, highlighting the need to understand the mechanisms governing their maintenance and reactivation. Emerging therapies targeting the OX40/OX40L axis and type 2 cytokines offer the potential to disrupt these memory reservoirs and move toward true disease modification, but critical questions remain regarding their ability to eliminate or reprogram memory cells, the optimal timing of intervention, and the biomarkers needed to personalize treatment. Advancing toward disease modification will require therapeutic strategies that effectively interrupt these latent immunological circuits, ultimately transforming the long-term management of AD.

## Figures and Tables

**Table 1:** Immune cells subtypes involved in atopic dermatitis pathogenesis and defining surface markers.

Cell Subtype	Surface Markers
**T-cells**	
**Tissue-Resident Memory T Cells (TRM)**	CD69, CD103, CCR4, CCR10, CLA
**CLA^+^ T cells**	CLA, HLA-DR, CD25, ICOS, CD40L
**Th2 cells**	CCR4^+^ CCR8^+^ CRTH2^+^ GATA3^+^ CD45RO^+^
**Th2A cells**	Th2 surface markerks + CRTH2^+^, CD161^+^, ST2^+^, CD27^−^, IL17RB, CRLF2
Th17 cells	CCR6^+^, CCR4^+^, CD161^+^, IL-23R^+^, RORγt^+^
Th22 cells	CCR10^+^, CCR6^+^, CCR4^+^, CLA^+^
Th1 cells	CXCR3^+^, CCR5^+^, T-bet^+^, CD45RO^+^
**B-cells**	
MBC2	CD23^hi^ IL4Rα^+^ IgG1^+^
**Innate immune cells**	
ILC2	CRTH2^+^ CD127^+^ CD161^+^ ST2^+^ IL17RB^+^ TSLPR^+^ CD81^+^
ILC3	CD117^+^ CD127^+^ RORγt^+^ NKp44^+^/^−^ CCR6^+^
IDEC	FcεRI^high^ CD1a^+^ CD11c^+^ CD206^+^ CD80/86^+^ HLA-DR^+^ CD207^−^
Langerhans cells	CD207^+^ CD1a^+^ HLA-DR^+^ EpCAM^+^ E-cadherin^+^ FcεRI^+^

Abbreviations used in this table are defined as follows: TRM, Tissue-Resident Memory T Cells; CLA, Cutaneous Lymphocyte-Associated Antigen; HLA-DR, Human Leukocyte Antigen–DR isotype; ICOS, Inducible T-cell COStimulator; CD40L, CD40 Ligand; CRTH2, Chemoattractant Receptor-Homologous molecule expressed on Th2 cells; GATA3, GATA Binding Protein 3; IL17RB, Interleukin-17 Receptor B; CRLF2, Cytokine Receptor-Like Factor 2; RORγt, RAR-Related Orphan Receptor Gamma t; T-bet, T-box Transcription Factor TBX21; MBC, Memory B Cell; ILC, Innate Lymphoid Cell; ILC2, Innate Lymphoid Cell type 2; ILC3, Innate Lymphoid Cell type 3; FcεRI, High-Affinity IgE Receptor; EpCAM, Epithelial Cell Adhesion Molecule. The main categories in the table are highlighted in bold for clarity.

## Data Availability

The data that support the findings of this study are available from the corresponding author upon reasonable request.

## Abbreviations

The following abbreviations are used in this manuscript:

AD	Atopic dermatitis
APC	Antigen-presenting cell
BCL	B-cell lymphoma
BCR	B-cell receptor
CCL	C–C motif chemokine ligand
CEIm	Clinical Research Ethics Committee
CLA	Cutaneous lymphocyte-associated antigen
DC	Dendritic cell
DLQI	Dermatology Life Quality Index
EASI	Eczema Area and Severity Index
IgE	Immunoglobulin E
IgG1	Immunoglobulin G1
IL	Interleukin
IRB	Institutional Review Board
MBC2	Type 2 memory B cells
NRS	Numeric Rating Scale

## References

[1] Czarnowicki T., Santamaria-Babí L.F., Guttman-Yassky E. (2017). Circulating CLA^+^ T Cells in Atopic Dermatitis and Their Possible Role as Peripheral Biomarkers. Allergy.

[2] Santamaria-Babí L.F. (2022). Atopic Dermatitis Pathogenesis: Lessons from Immunology. Dermatol. Pract. Concept..

[3] Thyssen J.P., Halling A.-S., Schmid-Grendelmeier P., Guttman-Yassky E., Silverberg J.I. (2023). Comorbidities of Atopic Dermatitis-What Does the Evidence Say?. J. Allergy Clin. Immunol..

[4] Berna-Rico E., Pérez-Garcia B., Gómez-de la Fuente E., Pérez-Bootello J., Abbad-Jaime de Aragon C., Neria F., Monge D., Domínguez-López R., Aranda C.J., Jaen P. (2026). Moderate-to-Severe Atopic Dermatitis and Systemic Corticosteroids Are Associated with Insulin Resistance and Metabolic Syndrome. JAAD Int..

[5] Berna-Rico E., Pérez-García B., Abbad-Jaime de Aragón C., Neria F., Monge D., Pérez-Bootello J., Gómez-De la Fuente E., Ortiz-De Frutos F.J., Fernández-Friera L., Solís J. (2025). Subclinical Atherosclerosis Is Increased in Moderate-to-Severe Atopic Dermatitis. J. Eur. Acad. Dermatol. Venereol. JEADV.

[6] Tsakok T., Marrs T., Mohsin M., Baron S., du Toit G., Till S., Flohr C. (2016). Does Atopic Dermatitis Cause Food Allergy? A Systematic Review. J. Allergy Clin. Immunol..

[7] Yamamoto-Hanada K., Kobayashi T., Mikami M., Williams H.C., Saito H., Saito-Abe M., Sato M., Irahara M., Miyaji Y., Ishikawa F. (2023). Enhanced Early Skin Treatment for Atopic Dermatitis in Infants Reduces Food Allergy. J. Allergy Clin. Immunol..

[8] Jacobson M.E., Seshadri R.S., Morimoto R., Grinich E., Haag C., Nguyen K., Simpson E.L. (2024). Early Intervention and Disease Modification in Atopic Dermatitis-the Current State of the Field and Barriers to Progress. J. Eur. Acad. Dermatol. Venereol. JEADV.

[9] de Jesús-Gil C., Sans-de SanNicolàs L., García-Jiménez I., Ferran M., Celada A., Chiriac A., Pujol R.M., Santamaria-Babí L.F. (2021). The Translational Relevance of Human Circulating Memory Cutaneous Lymphocyte-Associated Antigen Positive T Cells in Inflammatory Skin Disorders. Front. Immunol..

[10] Pham J.P., Wark K.J.L., Woods J., Frew J.W. (2023). Resident Cutaneous Memory T Cells: A Clinical Review of Their Role in Chronic Inflammatory Dermatoses and Potential as Therapeutic Targets. Br. J. Dermatol..

[11] Bangert C., Rindler K., Krausgruber T., Alkon N., Thaler F.M., Kurz H., Ayub T., Demirtas D., Fortelny N., Vorstandlechner V. (2021). Persistence of Mature Dendritic Cells, TH2A, and Tc2 Cells Characterize Clinically Resolved Atopic Dermatitis under IL-4Rα Blockade. Sci. Immunol..

[12] Chen L., Shen Z. (2020). Tissue-Resident Memory T Cells and Their Biological Characteristics in the Recurrence of Inflammatory Skin Disorders. Cell. Mol. Immunol..

[13] Huang Z., Chu M., Chen X., Wang Z., Jiang L., Ma Y., Wang Y. (2022). Th2A Cells: The Pathogenic Players in Allergic Diseases. Front. Immunol..

[14] Santamaria-Babí L.F. (2004). CLA^+^ T Cells in Cutaneous Diseases. Eur. J. Dermatol..

[15] Nicolàs L.S.S., Czarnowicki T., Akdis M., Pujol R.M., Lozano-Ojalvo D., Leung D.Y.M., Guttman-Yassky E., Santamaria-Babí L.F. (2024). CLA^+^ Memory T Cells in Atopic Dermatitis. Allergy.

[16] Wollenberg A., Weidinger S., Worm M., Bieber T. (2021). Tralokinumab in Atopic Dermatitis. JDDG J. Dtsch. Dermatol. Ges..

[17] Nitulescu G., Olaru O.T., Andrei C., Nitulescu G.M., Zanfirescu A. (2025). Targeting Intracellular Pathways in Atopic Dermatitis with Small Molecule Therapeutics. Curr. Issues Mol. Biol..

[18] Guttman-Yassky E., Blauvelt A., Eichenfield L.F., Paller A.S., Armstrong A.W., Drew J., Gopalan R., Simpson E.L. (2020). Efficacy and Safety of Lebrikizumab, a High-Affinity Interleukin 13 Inhibitor, in Adults with Moderate to Severe Atopic Dermatitis: A Phase 2b Randomized Clinical Trial. JAMA Dermatol..

[19] Ständer S., Yosipovitch G., Legat F.J., Reich A., Paul C., Simon D., Naldi L., Metz M., Tsianakas A., Pink A. (2025). Efficacy and Safety of Nemolizumab in Patients with Moderate to Severe Prurigo Nodularis: The OLYMPIA 1 Randomized Clinical Phase 3 Trial. JAMA Dermatol..

[20] Chen M., Wen X., Liu J., Yang G., Li Q., Jiang Z., Zhang X., Cai Z., Zhang L. (2025). Recurrence and Influencing Factors of Moderate-to-Severe Atopic Dermatitis after Dupilumab Withdrawal: A Retrospective Cohort Analysis. Front. Med..

[21] Silverberg J.I., Wollenberg A., Stein Gold L., Del Rosso J., Yosipovitch G., Lio P., Carrascosa J.-M., Gallo G., Ding Y., Xu Z. (2024). Patients with Moderate-to-Severe Atopic Dermatitis Maintain Stable Response with No or Minimal Fluctuations with 1 Year of Lebrikizumab Treatment. Dermatol. Ther..

[22] Guttman-Yassky E., Silverberg J.I., Thaçi D., Papp K.A., Ständer S., Beck L.A., Kim B.S., Hu X., Liu J., Calimlim B.M. (2023). Upadacitinib Treatment Withdrawal and Retreatment in Patients with Moderate-to-Severe Atopic Dermatitis: Results from a Phase 2b, Randomized, Controlled Trial. J. Eur. Acad. Dermatol. Venereol. JEADV.

[23] Wu H., Zhu J., Yang N., Ji X., Li Z., Zhou Y., Xu Q., Ye Y., Bai Z., Wang J. (2025). Atopic Dermatitis Relapse after Treatment Discontinuation and Predictive Factors for Relapse: JAK1 Inhibitors versus Dupilumab. JDDG J. Dtsch. Dermatol. Ges..

[24] Zhu J., Wu H., Ye Y., Xu Q., Shao J., Bai Z., Zhou Y., Li Z., Liu J., Li Z. (2024). Efficacy, Safety, and Early Relapse After Cessation of Upadacitinib Versus Dupilumab in Adolescents with Moderate-to-Severe Atopic Dermatitis^®^: A Real-World Study in China. Dermatitis.

[25] Fukushima-Nomura A., Kawasaki H., Yashiro K., Obata S., Tanese K., Ebihara T., Saeki H., Etoh T., Hasegawa T., Yazaki J. (2025). An Unbiased Tissue Transcriptome Analysis Identifies Potential Markers for Skin Phenotypes and Therapeutic Responses in Atopic Dermatitis. Nat. Commun..

[26] Seremet T., Di Domizio J., Girardin A., Yatim A., Jenelten R., Messina F., Saidoune F., Schlapbach C., Bogiatzi S., Minisini F. (2024). Immune Modules to Guide Diagnosis and Personalized Treatment of Inflammatory Skin Diseases. Nat. Commun..

[27] Croft M., Esfandiari E., Chong C., Hsu H., Kabashima K., Kricorian G., Warren R.B., Wollenberg A., Guttman-Yassky E. (2024). OX40 in the Pathogenesis of Atopic Dermatitis-A New Therapeutic Target. Am. J. Clin. Dermatol..

[28] Furue M., Ulzii D., Vu Y.H., Tsuji G., Kido-Nakahara M., Nakahara T. (2019). Pathogenesis of Atopic Dermatitis: Current Paradigm. Iran. J. Immunol. IJI.

[29] Howell M.D., Kim B.E., Gao P., Grant A.V., Boguniewicz M., DeBenedetto A., Schneider L., Beck L.A., Barnes K.C., Leung D.Y.M. (2009). Cytokine Modulation of Atopic Dermatitis Filaggrin Skin Expression. J. Allergy Clin. Immunol..

[30] Cesare A.D., Meglio P.D., Nestle F.O. (2008). A Role for Th17 Cells in the Immunopathogenesis of Atopic Dermatitis?. J. Investig. Dermatol..

[31] Gutowska-Owsiak D., Schaupp A.L., Salimi M., Selvakumar T.A., McPherson T., Taylor S., Ogg G.S. (2012). IL-17 Downregulates Filaggrin and Affects Keratinocyte Expression of Genes Associated with Cellular Adhesion. Exp. Dermatol..

[32] Cho K.-A., Suh J.W., Lee K.H., Kang J.L., Woo S.-Y. (2012). IL-17 and IL-22 Enhance Skin Inflammation by Stimulating the Secretion of IL-1β by Keratinocytes via the ROS-NLRP3-Caspase-1 Pathway. Int. Immunol..

[33] He H., Suryawanshi H., Morozov P., Gay-Mimbrera J., Del Duca E., Kim H.J., Kameyama N., Estrada Y., Der E., Krueger J.G. (2020). Single-Cell Transcriptome Analysis of Human Skin Identifies Novel Fibroblast Subpopulation and Enrichment of Immune Subsets in Atopic Dermatitis. J. Allergy Clin. Immunol..

[34] Weidinger S., Beck L.A., Bieber T., Kabashima K., Irvine A.D. (2018). Atopic Dermatitis. Nat. Rev. Dis. Primer.

[35] Czarnowicki T., Gonzalez J., Shemer A., Malajian D., Xu H., Zheng X., Khattri S., Gilleaudeau P., Sullivan-Whalen M., Suárez-Fariñas M. (2015). Severe Atopic Dermatitis Is Characterized by Selective Expansion of Circulating TH2/TC2 and TH22/TC22, but Not TH17/TC17, Cells within the Skin-Homing T-Cell Population. J. Allergy Clin. Immunol..

[36] Bakker D.S., van der Wal M.M., Heeb L.E.M., Giovannone B., Asamoah M., Delemarre E.M., Drylewicz J., Nierkens S., Boyman O., de Bruin-Weller M.S. (2021). Early and Long-Term Effects of Dupilumab Treatment on Circulating T-Cell Functions in Patients with Moderate-to-Severe Atopic Dermatitis. J. Investig. Dermatol..

[37] Elsner J.S., Carlsson M., Stougaard J.K., Nygaard U., Buchner M., Fölster-Holst R., Hvid M., Vestergaard C., Deleuran M., Deleuran B. (2020). The OX40 Axis Is Associated with Both Systemic and Local Involvement in Atopic Dermatitis. Acta Derm. Venereol..

[38] Vu T.T., Koguchi-Yoshioka H., Watanabe R. (2021). Skin-Resident Memory T Cells: Pathogenesis and Implication for the Treatment of Psoriasis. J. Clin. Med..

[39] Karlen H., Yousefi S., Simon H.-U., Simon D. (2020). IL-15 Expression Pattern in Atopic Dermatitis. Int. Arch. Allergy Immunol..

[40] Werner S., Grose R. (2003). Regulation of Wound Healing by Growth Factors and Cytokines. Physiol. Rev..

[41] Letterio J.J., Roberts A.B. (1998). Regulation of Immune Responses by TGF-Beta. Annu. Rev. Immunol..

[42] Lee H.S., Kooshesh F., Sauder D.N., Kondo S. (1997). Modulation of TGF-Beta 1 Production from Human Keratinocytes by UVB. Exp. Dermatol..

[43] Chojnacka-Purpurowicz J., Owczarczyk-Saczonek A., Nedoszytko B. (2024). The Role of Gamma Delta T Lymphocytes in Physiological and Pathological Condition-Focus on Psoriasis, Atopic Dermatitis, Autoimmune Disorders, Cancer and Lymphomas. Int. J. Mol. Sci..

[44] Jin S.-P., Lee K., Bang Y.J., Jeon Y.-H., Jung S., Choi S.-J., Lee J.S., Kim J., Guttman-Yassky E., Park C.-G. (2024). Mapping the Immune Cell Landscape of Severe Atopic Dermatitis by Single-Cell RNA-Seq. Allergy.

[45] Silva-Filho J.L., Caruso-Neves C., Pinheiro A.A.S. (2014). IL-4: An Important Cytokine in Determining the Fate of T Cells. Biophys. Rev..

[46] Youngblood B., Hale J.S., Kissick H.T., Ahn E., Xu X., Wieland A., Araki K., West E.E., Ghoneim H.E., Fan Y. (2017). Effector CD8 T Cells Dedifferentiate into Long-Lived Memory Cells. Nature.

[47] Tsoi L.C., Rodriguez E., Degenhardt F., Baurecht H., Wehkamp U., Volks N., Szymczak S., Swindell W.R., Sarkar M.K., Raja K. (2019). Atopic Dermatitis Is an IL-13-Dominant Disease with Greater Molecular Heterogeneity Compared to Psoriasis. J. Investig. Dermatol..

[48] Ladle B.H., Li K.-P., Phillips M.J., Pucsek A.B., Haile A., Powell J.D., Jaffee E.M., Hildeman D.A., Gamper C.J. (2016). De Novo DNA Methylation by DNA Methyltransferase 3a Controls Early Effector CD8^+^ T-Cell Fate Decisions Following Activation. Proc. Natl. Acad. Sci. USA.

[49] Kersh E.N. (2006). Impaired Memory CD8 T Cell Development in the Absence of Methyl-CpG-Binding Domain Protein 2. J. Immunol..

[50] Lupancu T.J., Eivazitork M., Hamilton J.A., Achuthan A.A., Lee K.M.-C. (2023). CCL17/TARC in Autoimmunity and Inflammation—Not Just a T-Cell Chemokine. Immunol. Cell Biol..

[51] Vulcano M., Albanesi C., Stoppacciaro A., Bagnati R., D’Amico G., Struyf S., Transidico P., Bonecchi R., Del Prete A., Allavena P. (2001). Dendritic Cells as a Major Source of Macrophage-Derived Chemokine/CCL22 in Vitro and in Vivo. Eur. J. Immunol..

[52] Günther C., Bello-Fernandez C., Kopp T., Kund J., Carballido-Perrig N., Hinteregger S., Fassl S., Schwärzler C., Lametschwandtner G., Stingl G. (2005). CCL18 Is Expressed in Atopic Dermatitis and Mediates Skin Homing of Human Memory T Cells. J. Immunol..

[53] Mommert S., Schaper J.T., Schaper-Gerhardt K., Gutzmer R., Werfel T. (2021). Histamine Increases Th2 Cytokine-Induced CCL18 Expression in Human M2 Macrophages. Int. J. Mol. Sci..

[54] Scott T.E., Lewis C.V., Zhu M., Wang C., Samuel C.S., Drummond G.R., Kemp-Harper B.K. (2023). IL-4 and IL-13 Induce Equivalent Expression of Traditional M2 Markers and Modulation of Reactive Oxygen Species in Human Macrophages. Sci. Rep..

[55] Acacia de Sa Pinheiro A., Morrot A., Chakravarty S., Overstreet M., Bream J.H., Irusta P.M., Zavala F. (2007). IL-4 Induces a Wide-Spectrum Intracellular Signaling Cascade in CD8^+^ T Cells. J. Leukoc. Biol..

[56] Huang L.R., Chen F.L., Chen Y.T., Lin Y.M., Kung J.T. (2000). Potent Induction of Long-Term CD8^+^ T Cell Memory by Short-Term IL-4 Exposure during T Cell Receptor Stimulation. Proc. Natl. Acad. Sci. USA.

[57] Riou C., Dumont A.R., Yassine-Diab B., Haddad E.K., Sekaly R.-P. (2006). IL-4 Influences the Differentiation and the Susceptibility to Activation-Induced Cell Death of Human Naive CD8^+^ T Cells. Int. Immunol..

[58] Schärli S., Luther F., Di Domizio J., Hillig C., Radonjic-Hoesli S., Thormann K., Simon D., Rønnstad A.T.M., Ruge I.F., Fritz B.G. (2025). IL-9 Sensitizes Human TH2 Cells to Proinflammatory IL-18 Signals in Atopic Dermatitis. J. Allergy Clin. Immunol..

[59] Wambre E., Bajzik V., DeLong J.H., O’Brien K., Nguyen Q.-A., Speake C., Gersuk V.H., DeBerg H.A., Whalen E., Ni C. (2017). A Phenotypically and Functionally Distinct Human TH2 Cell Subpopulation Is Associated with Allergic Disorders. Sci. Transl. Med..

[60] Tanei R., Hasegawa Y. (2022). Immunological Pathomechanisms of Spongiotic Dermatitis in Skin Lesions of Atopic Dermatitis. Int. J. Mol. Sci..

[61] Gandhi N.A., Bennett B.L., Graham N.M.H., Pirozzi G., Stahl N., Yancopoulos G.D. (2016). Targeting Key Proximal Drivers of Type 2 Inflammation in Disease. Nat. Rev. Drug Discov..

[62] Furue M. (2020). Regulation of Filaggrin, Loricrin, and Involucrin by IL-4, IL-13, IL-17A, IL-22, AHR, and NRF2: Pathogenic Implications in Atopic Dermatitis. Int. J. Mol. Sci..

[63] Jin M., Yoon J. (2018). From Bench to Clinic: The Potential of Therapeutic Targeting of the IL-22 Signaling Pathway in Atopic Dermatitis. Immune Netw..

[64] Czarnowicki T., He H., Krueger J.G., Guttman-Yassky E. (2019). Atopic Dermatitis Endotypes and Implications for Targeted Therapeutics. J. Allergy Clin. Immunol..

[65] Alkon N., Bauer W.M., Krausgruber T., Goh I., Griss J., Nguyen V., Reininger B., Bangert C., Staud C., Brunner P.M. (2022). Single-Cell Analysis Reveals Innate Lymphoid Cell Lineage Infidelity in Atopic Dermatitis. J. Allergy Clin. Immunol..

[66] Nedoszytko B., Lange M., Sokołowska-Wojdyło M., Renke J., Trzonkowski P., Sobjanek M., Szczerkowska-Dobosz A., Niedoszytko M., Górska A., Romantowski J. (2017). The Role of Regulatory T Cells and Genes Involved in Their Differentiation in Pathogenesis of Selected Inflammatory and Neoplastic Skin Diseases. Part I: Treg Properties and Functions. Postepy Dermatol. Alergol..

[67] Shevyrev D., Tereshchenko V. (2019). Treg Heterogeneity, Function, and Homeostasis. Front. Immunol..

[68] Sakaguchi S., Yamaguchi T., Nomura T., Ono M. (2008). Regulatory T Cells and Immune Tolerance. Cell.

[69] Liu Y., Wang H., Taylor M., Cook C., Martínez-Berdeja A., North J.P., Harirchian P., Hailer A.A., Zhao Z., Ghadially R. (2022). Classification of Human Chronic Inflammatory Skin Disease Based on Single-Cell Immune Profiling. Sci. Immunol..

[70] Chapoval S., Dasgupta P., Dorsey N.J., Keegan A.D. (2010). Regulation of the T Helper Cell Type 2 (Th2)/T Regulatory Cell (Treg) Balance by IL-4 and STAT6. J. Leukoc. Biol..

[71] Dardalhon V., Awasthi A., Kwon H., Galileos G., Gao W., Sobel R.A., Mitsdoerffer M., Strom T.B., Elyaman W., Ho I.-C. (2008). IL-4 Inhibits TGF-Beta-Induced Foxp3^+^ T Cells and, Together with TGF-Beta, Generates IL-9+ IL-10+ Foxp3^−^ Effector T Cells. Nat. Immunol..

[72] Silverberg J.I., Rosmarin D., Chovatiya R., Bieber T., Schleicher S., Beck L., Gooderham M., Chaudhry S., Fanton C., Yu D. (2024). The Regulatory T Cell-Selective Interleukin-2 Receptor Agonist Rezpegaldesleukin in the Treatment of Inflammatory Skin Diseases: Two Randomized, Double-Blind, Placebo-Controlled Phase 1b Trials. Nat. Commun..

[73] Fanton C., Zalevsky J. (2024). Recent Patents in Allergy and Immunology: The Interleukin-2 Receptor Pathway Agonist Rezpegaldesleukin (REZPEG) for the Rescue of Regulatory T Cells in Chronic Inflammatory and Autoimmune Diseases. Allergy.

[74] Ota M., Hoehn K.B., Fernandes-Braga W., Ota T., Aranda C.J., Friedman S., Miranda-Waldetario M.G.C., Redes J., Suprun M., Grishina G. (2024). CD23^+^IgG1^+^ Memory B Cells Are Poised to Switch to Pathogenic IgE Production in Food Allergy. Sci. Transl. Med..

[75] Saunders S.P., Ma E.G.M., Aranda C.J., Curotto de Lafaille M.A. (2019). Non-Classical B Cell Memory of Allergic IgE Responses. Front. Immunol..

[76] Palomares Ó., Sánchez-Ramón S., Dávila I., Prieto L., Pérez de Llano L., Lleonart M., Domingo C., Nieto A. (2017). dIvergEnt: How IgE Axis Contributes to the Continuum of Allergic Asthma and Anti-IgE Therapies. Int. J. Mol. Sci..

[77] Aranda C.J., Gonzalez-Kozlova E., Saunders S.P., Fernandes-Braga W., Ota M., Narayanan S., He J.-S., Del Duca E., Swaroop B., Gnjatic S. (2023). IgG Memory B Cells Expressing IL4R and FCER2 Are Associated with Atopic Diseases. Allergy.

[78] Starrenburg M.E., Bel Imam M., Lopez J.F., Buergi L., Nguyen N.T., Nouwen A.E.M., Arends N.J.T., Caspers P.J., Akdis M., Pasmans S.G.M.A. (2024). Dupilumab Treatment Decreases MBC2s, Correlating with Reduced IgE Levels in Pediatric Atopic Dermatitis. J. Allergy Clin. Immunol..

[79] Werfel T., Heratizadeh A., Niebuhr M., Kapp A., Roesner L.M., Karch A., Erpenbeck V.J., Lösche C., Jung T., Krug N. (2015). Exacerbation of Atopic Dermatitis on Grass Pollen Exposure in an Environmental Challenge Chamber. J. Allergy Clin. Immunol..

[80] Novak N., Valenta R., Bohle B., Laffer S., Haberstok J., Kraft S., Bieber T. (2004). FcepsilonRI Engagement of Langerhans Cell-like Dendritic Cells and Inflammatory Dendritic Epidermal Cell-like Dendritic Cells Induces Chemotactic Signals and Different T-Cell Phenotypes in Vitro. J. Allergy Clin. Immunol..

[81] Tham E.H., Rajakulendran M., Lee B.W., Van Bever H.P.S. (2020). Epicutaneous Sensitization to Food Allergens in Atopic Dermatitis: What Do We Know?. Pediatr. Allergy Immunol..

[82] Esaki H., Brunner P.M., Renert-Yuval Y., Czarnowicki T., Huynh T., Tran G., Lyon S., Rodriguez G., Immaneni S., Johnson D.B. (2016). Early-Onset Pediatric Atopic Dermatitis Is TH2 but Also TH17 Polarized in Skin. J. Allergy Clin. Immunol..

[83] Ito T., Wang Y.-H., Duramad O., Hori T., Delespesse G.J., Watanabe N., Qin F.X.-F., Yao Z., Cao W., Liu Y.-J. (2005). TSLP-Activated Dendritic Cells Induce an Inflammatory T Helper Type 2 Cell Response through OX40 Ligand. J. Exp. Med..

[84] Zhou B., Comeau M.R., De Smedt T., Liggitt H.D., Dahl M.E., Lewis D.B., Gyarmati D., Aye T., Campbell D.J., Ziegler S.F. (2005). Thymic Stromal Lymphopoietin as a Key Initiator of Allergic Airway Inflammation in Mice. Nat. Immunol..

[85] Pan Y., Hochgerner M., Cichoń M.A., Benezeder T., Bieber T., Wolf P. (2025). Langerhans Cells: Central Players in the Pathophysiology of Atopic Dermatitis. J. Eur. Acad. Dermatol. Venereol..

[86] von Bubnoff D., Novak N., Kraft S., Bieber T. (2003). The Central Role of FcepsilonRI in Allergy. Clin. Exp. Dermatol..

[87] Maurer D., Ebner C., Reininger B., Petzelbauer P., Fiebiger E., Stingl G. (1997). Mechanisms of Fc Epsilon RI-IgE-Facilitated Allergen Presentation by Dendritic Cells. Adv. Exp. Med. Biol..

[88] Martín-Cruz L., Sevilla-Ortega C., Angelina A., Domínguez-Andrés J., Netea M.G., Subiza J.L., Palomares O. (2023). From Trained Immunity in Allergy to Trained Immunity-Based Allergen Vaccines. Clin. Exp. Allergy.

[89] Martinez-Gonzalez I., Mathä L., Steer C.A., Ghaedi M., Poon G.F.T., Takei F. (2016). Allergen-Experienced Group 2 Innate Lymphoid Cells Acquire Memory-like Properties and Enhance Allergic Lung Inflammation. Immunity.

[90] Nishimura M., Imai Y., Matsushima Y., Yamanaka K. (2025). Comprehensive Analysis of Type 2 Innate Lymphoid Cell (ILC2) Heterogeneity and Identification of CD81 as a Marker for Pathogenic IL-13^+^ ILC2s in Atopic Dermatitis Skin. Allergy.

[91] Jia H., Wan H., Zhang D. (2023). Innate Lymphoid Cells: A New Key Player in Atopic Dermatitis. Front. Immunol..

[92] To T.T., Oparaugo N.C., Kheshvadjian A.R., Nelson A.M., Agak G.W. (2024). Understanding Type 3 Innate Lymphoid Cells and Crosstalk with the Microbiota: A Skin Connection. Int. J. Mol. Sci..

[93] Netea M.G., Domínguez-Andrés J., Barreiro L.B., Chavakis T., Divangahi M., Fuchs E., Joosten L.A.B., van der Meer J.W.M., Mhlanga M.M., Mulder W.J.M. (2020). Defining Trained Immunity and Its Role in Health and Disease. Nat. Rev. Immunol..

[94] Song R., Zhang H., Liang Z. (2024). Research Progress in OX40/OX40L in Allergic Diseases. Int. Forum Allergy Rhinol..

[95] Guttman-Yassky E., Croft M., Geng B., Rynkiewicz N., Lucchesi D., Peakman M., van Krinks C., Valdecantos W., Xing H., Weidinger S. (2024). The Role of OX40 Ligand/OX40 Axis Signalling in Atopic Dermatitis. Br. J. Dermatol..

[96] Guttman-Yassky E., Simpson E., Esfandiari E., Mano H., Bauer J., Charuworn P., Kabashima K. (2025). Rocatinlimab: A Novel T-Cell Rebalancing Therapy Targeting the OX40 Receptor in Atopic Dermatitis. Dermatol. Ther..

[97] Weidinger S., Bieber T., Cork M.J., Reich A., Wilson R., Quaratino S., Stebegg M., Brennan N., Gilbert S., O’Malley J.T. (2023). Safety and Efficacy of Amlitelimab, a Fully Human Nondepleting, Noncytotoxic Anti-OX40 Ligand Monoclonal Antibody, in Atopic Dermatitis: Results of a Phase IIa Randomized Placebo-Controlled Trial. Br. J. Dermatol..

[98] de Oliveira H.M., Gallo Ruelas M., Viana Diaz C.A., Ghanem L., Lima de Oliveira L.M. (2025). Safety and Efficacy of Anti-OX40 Therapies in Atopic Dermatitis: A Systematic Review and Meta-Analysis. Dermatitis.

[99] Guttman-Yassky E., Simpson E.L., Reich K., Kabashima K., Igawa K., Suzuki T., Mano H., Matsui T., Esfandiari E., Furue M. (2023). An Anti-OX40 Antibody to Treat Moderate-to-Severe Atopic Dermatitis: A Multicentre, Double-Blind, Placebo-Controlled Phase 2b Study. Lancet.

[100] Morant A.V., Vestergaard H.T., Lassen A.B., Navikas V. (2020). US, EU, and Japanese Regulatory Guidelines for Development of Drugs for Treatment of Alzheimer’s Disease: Implications for Global Drug Development. Clin. Transl. Sci..

[101] Morant A.V., Jagalski V., Vestergaard H.T. (2019). Labeling of Disease-Modifying Therapies for Neurodegenerative Disorders. Front. Med..

[102] Fukuie T., Hirakawa S., Narita M., Nomura I., Matsumoto K., Tokura Y., Ohya Y. (2016). Potential Preventive Effects of Proactive Therapy on Sensitization in Moderate to Severe Childhood Atopic Dermatitis: A Randomized, Investigator-Blinded, Controlled Study. J. Dermatol..

[103] Lin T.-L., Fan Y.-H., Fan K.-S., Juan C.-K., Chen Y.-J., Wu C.-Y. (2024). Reduced Atopic March Risk in Pediatric Atopic Dermatitis Patients Prescribed Dupilumab versus Conventional Immunomodulatory Therapy: A Population-Based Cohort Study. J. Am. Acad. Dermatol..

[104] Silverberg J.I., Guttman-Yassky E., Thaçi D., Irvine A.D., Stein Gold L., Blauvelt A., Simpson E.L., Chu C.-Y., Liu Z., Gontijo Lima R. (2023). Two Phase 3 Trials of Lebrikizumab for Moderate-to-Severe Atopic Dermatitis. N. Engl. J. Med..

[105] Blauvelt A., Thyssen J.P., Guttman-Yassky E., Bieber T., Serra-Baldrich E., Simpson E., Rosmarin D., Elmaraghy H., Meskimen E., Natalie C.R. (2023). Efficacy and Safety of Lebrikizumab in Moderate-to-Severe Atopic Dermatitis: 52-Week Results of Two Randomized Double-Blinded Placebo-Controlled Phase III Trials. Br. J. Dermatol..

[106] Liñán-Barroso J.-M., Hernández-Rodríguez J.-C., Ruiz-Villaverde R., Galán Gutiérrez M., Navarro-Triviño F., Domínguez Cruz J., Armario-Hita J.-C., Pereyra-Rodriguez J.-J. (2025). Definition of the Concept of Super-Responders in Atopic Dermatitis: A Spanish Delphi Consensus. Acta Derm. Venereol..

[107] Schäkel K., Reich K., Asadullah K., Pinter A., Jullien D., Weisenseel P., Paul C., Gomez M., Wegner S., Personke Y. (2023). Early Disease Intervention with Guselkumab in Psoriasis Leads to a Higher Rate of Stable Complete Skin Clearance (‘clinical Super Response’): Week 28 Results from the Ongoing Phase IIIb Randomized, Double-Blind, Parallel-Group, GUIDE Study. J. Eur. Acad. Dermatol. Venereol. JEADV.

[108] Silverberg J.I., Simpson E.L., Pink A.E., Weidinger S., Chan G., Biswas P., Clibborn C., Güler E. (2025). Switching from Dupilumab to Abrocitinib in Patients with Moderate-to-Severe Atopic Dermatitis: A Post Hoc Analysis of Efficacy After Treatment with Dupilumab in JADE DARE. Dermatol. Ther..

[109] Alegre-Bailo A., Sánchez-Gilo A., Román Mendoza N.M., Mateos-Rico J.J., Vicente-Martín F.J. (2023). Tralokinumab Treatment in Atopic Dermatitis: Depicting Super-Responders. J. Dermatol..

[110] Mastorino L., Mendes-Bastos P., Ribero S., Ortoncelli M. (2025). The Need for a Shared Definition of the Atopic Dermatitis “super Responder” in the “Treat-to-Target” Era. Clin. Exp. Dermatol..

